# Willingness to use HIV pre-exposure prophylaxis among gay men, other men who have sex with men and transgender women in Myanmar

**DOI:** 10.7448/IAS.20.1.21885

**Published:** 2017-07-26

**Authors:** Bridget L. Draper, Zaw Min Oo, Zaw Win Thein, Poe Poe Aung, Vanessa Veronese, Claire Ryan, Myo Thant, Chad Hughes, Mark Stoové

**Affiliations:** ^a^ Department of Disease Elimination, Burnet Institute, Melbourne, Australia; ^b^ Department of International Development, Burnet Institute, Yangon, Myanmar; ^c^ Department of Epidemiology and Preventive Medicine, Monash University, Melbourne, Australia; ^d^ Yangon Regional Public Health Department, Yangon, Myanmar

**Keywords:** PrEP, MSM, Burma, acceptability, awareness, transgender women

## Abstract

**Introduction**: HIV pre-exposure prophylaxis (PrEP) has emerged as a key component of contemporary HIV combination prevention strategies. To explore the local suitability of PrEP, country-specific acceptability studies are needed to inform potential PrEP implementation. In the context of Myanmar, in addition to resource constraints, HIV service access by gay men, other men who have sex with men, and transgender women (GMT) continues to be constrained by legislative and community stigma and marginalization. We aimed to determine PrEP acceptability among GMT in Myanmar and explore the factors associated with willingness to use PrEP.

**Methods**: GMT were recruited in Yangon and Mandalay through local HIV prevention outreach programmes in November and December 2014. Quantitative surveys were administered by trained peer educators and collected data on demographics, sexual risk, testing history and PrEP acceptability. A modified six-item PrEP acceptability scale classified self-reported HIV undiagnosed GMT as willing to use PrEP. Multivariable logistic regression identified factors associated with willingness to use PrEP.

**Results**: Among 434 HIV undiagnosed GMT, PrEP awareness was low (5%). PrEP acceptability was high, with 270 (62%) GMT classified as willing to use PrEP. GMT recruited in Mandalay (adjusted odds ratio (aOR) = 1.79; 95%CI = 1.05–3.03), who perceived themselves as likely to become HIV positive (aOR = 1.82; 95%CI = 1.10–3.02), who had more than one recent regular partner (aOR = 2.94; 95%CI = 1.41–6.14), no regular partners (aOR = 2.05; 95%CI = 1.10–3.67), more than five casual partners (aOR = 2.05; 95%CI = 1.06–3.99) or no casual partners (aOR = 2.25; 95%CI = 1.23–4.11) were more likely to be willing to use PrEP. The association between never or only occasionally using condoms with casual partners and willingness to use PrEP was marginally significant (aOR = 2.02; 95%CI = 1.00–4.10). GMT who reported concern about side effects and long-term use of PrEP were less likely (aOR = 0.35; 95%CI = 0.21–0.59) to be willing to use PrEP.

**Conclusions**: This is the first study to assess PrEP acceptability in Myanmar. Findings suggest PrEP is an acceptable prevention option among GMT in Myanmar, providing they are not required to pay for it. Implementation/demonstration projects are needed to explore the feasibility and cost-effectiveness of PrEP as a prevention option for GMT in Myanmar.

## Introduction

HIV pre-exposure prophylaxis (PrEP) – the use of antiretroviral drugs by HIV negative individuals to prevent the acquisition of HIV – is increasingly recognized as a fundamental component of combination HIV prevention strategies [1,2]. Clinical trials, open-label extension studies and demonstration projects have shown PrEP to be safe and highly effective in reducing the risk of HIV acquisition among men who have sex with men (MSM) [3–7], heterosexuals [8,9] and people who inject drugs [10]. Extending earlier recommendations focussed on MSM [1], in 2015 the World Health Organization (WHO) recommended PrEP be offered as an additional prevention option to all those at substantial risk of HIV [11].

In light of demonstrated prevention effectiveness, studies focussing on the local acceptability of PrEP among key populations are needed to inform implementation. Studies assessing the willingness to use PrEP among MSM have been conducted in both high- and low-income countries. In high-income countries, PrEP acceptability among MSM has varied considerably, from 40%–60% in the US, Canada and the UK [12–14] to approximately 25% in Australia [15] and 13% in the Netherlands [16]. PrEP acceptability among MSM has generally been higher in most studies conducted in low- and middle-income countries with the majority of MSM classified as willing to use PrEP in China, Brazil, Peru and Kenya [17–22]. However, lower levels of PrEP acceptability (around 40%) have been reported in Thailand [23–25]. Factors associated with increased willingness to use PrEP have included younger age [14,26], sexual risk behaviours [14,16,19,25–27], sexual relationships with HIV-positive partners [12,16,20,26] and self-perceived HIV risk [14,15,20,26]. Concerns about side effects and long-term use of PrEP have consistently been associated with reduced willingness to use PrEP and are a barrier to the uptake of PrEP [15,16,22,26].

Broader issues associated with health systems and enabling environments for HIV prevention also influence the feasibility and acceptability of PrEP programmes. Stigma and discrimination experienced by people living with HIV and key populations in healthcare settings more broadly are major barriers to HIV prevention [28–30]. In particular, issues of trust, accessibility and confidentiality in healthcare settings have been highlighted as important to supporting medication adherence and the effectiveness of PrEP [28].

Given the variations in local HIV epidemiological, health systems and sociopolitical environments globally, country-specific studies are required to inform the potential implementation of PrEP [31]. In Myanmar, MSM and transgender women continue to be disproportionately affected by HIV with prevalence of HIV among MSM in Yangon estimated to be at 27% in 2015 [32]. MSM also face considerable discrimination in a country where male-to-male sex remains criminalized [33,34], with the latest National Strategic Plan on HIV and AIDS emphasizing the importance of tailored, integrated and peer-involved HIV prevention and care service models to help overcome barriers to HIV prevention and care service access [35].

This study aimed to assess PrEP acceptability and associated factors among GMT recruited through HIV peer outreach programmes in Myanmar.

## Methods

### Recruitment and data collection

Cross-sectional survey data were collected from GMT recruited through the Burnet Institute and Myanmar Business Coalition on AIDS (MBCA) HIV prevention outreach programme. The survey was administered by trained peer-researchers using a password-protected electronic tablet in November and December 2014 using time- and venue-based convenience and snow-ball sampling. GMT were identified by HIV prevention peer outreach workers operating in public cruising areas in Yangon and Mandalay. Interested participants were given a study card listing potential times and locations to complete the survey. Up to three additional study cards were provided to potential participants to refer other GMT into the study. Approximately 15% of those given a study card did not attend to complete the study survey at the agreed upon time and place. Data collection occurred at mutually acceptable locations to ensure participant safety. Participants were eligible if they were at least 18 years old, self-reported as GMT, presented with a study card and were able to provide informed consent.

The study was approved by the Alfred Health Ethics Committee (Project Number: 445/14) and the Department of Medical Research (Lower Myanmar) Ethical Review Committee.

### Measures

The survey covered demographic characteristics, sexual risk behaviours, HIV status, HIV testing history, barriers to regular HIV testing and attitudes towards condoms and PrEP. Self-reported HIV status was dichotomized into diagnosed or undiagnosed (HIV negative/unsure/never tested).

The primary outcome for this study was willingness to use PrEP. All participants were asked about their awareness of PrEP. Participants were then presented with a standardized description of PrEP that explained recommended frequency (daily) and adherence, general HIV prevention effectiveness, safety (including reference to mild, temporary side effects), and that PrEP is approved by the WHO, but not yet approved or available in Myanmar.

Willingness to use PrEP was then assessed among HIV undiagnosed GMT using a modified version of a validated seven-item scale [26]. To account for the likelihood that future PrEP programmes in Myanmar would be provided at no cost (most HIV services in Myanmar are funded by international donors and HIV testing and prevention services are provided at no cost or offer small incentives to key populations), we removed one item from the scale referring to willingness to pay for PrEP. The willingness to use PrEP scale asks participants to respond to a series of statements about their likelihood of using PrEP on a Likert scale (1 = strongly disagree to 5 = strongly agree) with participants’ mean score across six items calculated. Consistent with the original scale classification procedure, a mean score equal to or greater than 4 was classified as willingness to use PrEP and scores below 4 were classified as not willing to use or neutral about PrEP.

Exposures were chosen based on previous research and knowledge of the local context. These included a range of variables measuring demographics, sexual behaviours and attitudes. Demographic variables included age, sexual identity (classified using locally accepted labels *Apone*, *Apwint* and *Thange*, see below), living situation and income. Sexual behaviour and health-seeking variables included the number of regular and casual male sex partners in the past three months (classified as “No regular male partners” “One regular male partner” and “More than one regular male partner” and “No casual male partners”, “2–5 casual male partners” and “5+ casual male partners” respectively), condom use with these partner types in the past three months (dichotomised as “Often/Always” and “Never/Occasionally”), HIV status of regular male partner(s) (classified as “Negative”, “Positive/suspected positive” and “Don’t know”) and time since last HIV test (classified as “In the last 6 months”, “6 months to 2 years ago” and “>2 years ago/Never tested”). Attitudinal items included a personal experience using condoms scale [26], a confidence discussing condom use scale [26], self-perceived risk of HIV (dichotomised as “Likely to become HIV positive” and “Not likely to become HIV positive”) and concern about using PrEP because of side effects and long-term use (dichotomized as “Concerned about using PrEP” and “Not concerned about using PrEP”). Gender and sexual identities in Myanmar do not mirror those generally seen in Western cultures. In this study, we refer to the key population as gay men, other men who have sex with men and transgender women in an effort to describe the grouping of locally accepted labels of sub-groups of men who have sex with men, which may also include people who would identify as transgender women in Western cultures. Definitions of these gender and sexual identities continue to evolve in Myanmar [36]. We define the labels referred to in this article as follows: *Apwint* are biological males who identify openly as feminine and openly disclose same-sex preferences, they may also include people who would identify as transgender women in Western cultures; *Apone* are men who have a masculine presentation, and in some spheres or circumstances may disclose same-sex behaviour and identify as gay, while in others are likely to conceal, or deny their same-sex preferences, and *Thange* are masculine, heterosexual-identifying men who conform to heteronormative expectations, and may engage in sexual relationships with women, while also engaging in incidental sex with other men [36].

### Data analysis

Univariable descriptive statistics present sample characteristics and agreement with specific willingness to use PrEP scale items. Multivariable logistic regression determined factors associated with willingness to use PrEP. To avoid case-wise dropping of variables, no sexual partner categories were included for both number of partners and condom use variables. To account for subsequent collinearity between these risk exposures, two adjusted models were constructed: Model 1 – inclusive of number of partners variables; Model 2 – inclusive of condom use variables. Variables included in the multivariable models were chosen based on relevant literature and knowledge of the local context. The outcome in both models was “willing to use PrEP”, defined as a score equal to or greater than 4 on the modified “willingness to use PrEP” scale. Statistical significance was defined as *p* < 0.05. Statistical analyses were performed using Stata (v13.1).

## Results

A total of 520 GMT completed the survey, 293 (56%) in Mandalay and 227 (44%) in Yangon. GMT who reported being HIV positive (*n* = 66, 13%), GMT who did not report their HIV status (*n* = 10, 2%) and GMT with missing data on any of the six willingness to use PrEP items (*n* = 10, 2%) were removed from subsequent analyses.

Table 1 describes the characteristics of the remaining 434 participants. On average, our participants were typically aged in their early 20s and almost half identified as *Thange*. The majority of participants reported living with their family, almost one third reported completing tertiary education and their median monthly income was 130,000 MMK, consistent with recent estimates of average national income (gross national income per capita) [37] and slightly above the minimum wage (110 000 MMK) [38]. Most GMT reported one or more regular male sex partners and two or more casual male sex partners in the past three months. More than half of GMT with regular partners reported often or always using condoms with these partners and almost three-quarters reported using condoms with casual partners often or always. Less one quarter of GMT were classified as having positive experiences using condoms but most reported feeling confident discussing condom use with partners. Two thirds reported having tested for HIV in the past six months. Approximately half reported perceiving themselves as likely to become HIV positive. Awareness of PrEP was low (5%). After having the attributes of PrEP explained, almost two thirds of GMT were classified as willing to use PrEP using the modified willingness to use PrEP scale.

**Table 1.** Participant characteristics of HIV undiagnosed men

	*N* = 434 *n* (%)
Location of interview	
Mandalay	253 (58)
Yangon	181 (42)
Age (years) (median, IQR)	23 (20, 28)
Sexual identity	
Apone	115 (27)
Apwint	103 (24)
Thange	211 (49)
Living situation	
Alone	27 (6)
Sex partner	56 (13)
Family	305 (70)
Non-related adults	45 (10)
Education level	
Primary school or below	34 (8)
High school	268 (62)
Tertiary education	131 (30)
Monthly income (MMK) (median, IQR)	130,000 (85,000, 200,000)
Number of regular male partners in past three months	
No regular male partners	195 (45)
1 regular male partner	164 (38)
>1 regular male partner	75 (17)
Condom use with regular male partners in past three months	(*n* = 234)
Often/always (>50%)	136 (58)
Never/Occasionally (<50%)	99 (42)
HIV status of regular male partner(s)	
Negative	145 (34)
Positive/suspect positive	17 (4)
Don’t know	267 (62)
Number of casual male partners in past three months	
No casual male partners	128 (30)
2–5 casual male partners	203 (48)
5+ casual male partners	91 (22)
Condom use with casual male partners in the past three months	(*n* = 294)
Often/always (>50%)	211 (72)
Never/occasionally (<50%)	81 (28)
At the last occasion you had anal sex with a casual male partner, how far in advance did you know you were going to have sex?	
One day or more	51 (12)
Less than an hour/a few hours	120 (29)
Did not know in advance	122 (29)
No anal sex with casual partners	128 (30)
Personal experience using condoms	
Negative/neutral	346 (82)
Positive	75 (18)
Confidence in discussing condom use with partners	
Not confident/neutral	129 (30)
Confident	301 (70)
Last tested for HIV	
In the last 6 months	291 (68)
6 months to 2 years ago	51 (12)
>2 years ago/never tested	89 (21)
Self-perceived risk of HIV	
Unlikely to become HIV+	221 (54)
Likely to become HIV+	185 (46)
If I was taking PrEP, I would be concerned that people will think I’m HIV positive	
Disagree	227 (53)
Agree	205 (47)
Concern about using PrEP because of side effects and long-term use	
Not concerned	251 (59)
Concerned	178 (41)
Awareness of PrEP	
Not aware	403 (95)
Aware	23 (5)
Willingness to use PrEP	
Willing to use PrEP	270 (62)
Not willing to use/neutral about PrEP	164 (38)

Missing data excluded, may not total *N* = 434.

Responses to each item of the willingness to use PrEP scale are detailed in Table 2. The proportion of participants in agreement with statements about willingness to take PrEP was consistently high in the modified scale (between 76% and 87%). As anticipated, agreement with the statement on willingness to pay for PrEP, which was part of the original scale, was relatively low (36%). As noted, this item was not included in the classification of willingness to use PrEP. The mean of participants’ mean score on the modified willingness to use PrEP scale was 3.88 (SD = 0.64).

**Table 2.** Responses to each item on the willingness to use PrEP scale

	Strongly disagree/disagree/neither agree nor disagree (scored 1–3)	Strongly agree/agree (scored 4–5)
I would be willing to take PrEP to prevent getting HIV	58 (13)	376 (87)
I would take pills before and after sex to prevent HIV	64 (15)	370 (85)
I would take a pill every day to prevent HIV	78 (18)	356 (82)
I am going to take PrEP as soon as it is available	78 (18)	356 (82)
I would never need to take PrEP (reverse scored)	72 (17)	362 (83)
I would take PrEP even if it wasn’t 100% effective	106 (14)	328 (76)
I would be willing to pay for PrEP*	273 (63)	158 (36)

*This item was excluded from the adapted outcome “willingness to use PrEP”.

Table 3 presents results from the two multivariable logistic regression models. Willingness to pay for PrEP was positively associated with being recruited in Mandalay, perceiving themselves as likely to become HIV positive and reporting in the past three months having had more than one regular male partner or reporting no regular male partners (compared with one regular male partner) and reporting five or more casual male partners or no casual male partners (compared with one casual partner). The association between reporting never or only occasionally using condoms with casual partners and willingness to use PrEP was marginally significant. Reporting concern about side effects and long-term use of PrEP was negatively associated with willingness to use PrEP.

**Table 3.** Factors associated with willingness to use PrEP

	Willingness to use PrEP	Model 1	Model 2
	(*N* = 434)*n* (%)	Adjusted odds ratio (95% confidence interval)	Adjusted odds ratio (95% confidence interval)
Location of interview			
Yangon	103 (57)	1	1
Mandalay	167 (66)	1.79 (1.05–3.03)*	1.81 (1.07–3.06)*
Age (years)		1.00 (0.97–1.04)	1.00 (0.97–1.04)
Sexual identity			
Apone	70 (61)	1	1
Apwint	68 (66)	1.06 (0.53–2.14)	1.34 (0.68–2.66)
Thange	130 (62)	1.10 (0.60–2.02)	1.14 (0.63–2.07)
Living situation			
Alone	13 (48)	1	1
Sex partner	32 (57)	1.37 (0.43 –4.32)	1.22 (0.39–3.79)
Family	192 (63)	1.28 (0.48–3.36)	1.39 (0.53–3.61)
Non-related adults	32 (71)	2.52 (0.74–8.60)	2.63 (0.78–8.82)
Monthly income			
Below median	125 (62)	1	1
Median & above	142 (63)	1.33 (0.82–2.16)	1.29 (0.80–2.08)
Number of regular male partners in past three months			
1 regular male partner	91 (55)	1	-
>1 regular male partner	56 (75)	2.94 (1.41–6.14)**	-
No regular male partners	123 (63)	2.01 (1.10–3.67)*	
Condom use with regular male partners in past three months			
Often/always (>50%)	84 (62)	–	1
Never/occasionally (<50%)	61 (62)	–	0.91 (0.45–1.84)
No regular male partners	123 (63)		1.23 (0.69–2.19)
HIV status of regular male partner(s)			
Negative	88 (61)	1	1
Positive/suspect positive	12 (71)	1.29 (0.27–6.13)	1.30 (0.28–6.03)
Don’t know	168 (63)	1.10 (0.63–1.90)	1.21 (0.70–2.09)
Number of casual male partners in past three months			
2−5 casual male partners	119 (59)	1	-
5+ casual male partners	60 (66)	2.05 (1.06–3.99)*	-
No casual male partners	84 (66)	2.25 (1.23–4.11)*	
Condom use with casual male partners in past three months			
Often/always (>50%)	127 (60)	–	1
Never/occasionally (<50%)	50 (62)	–	2.02 (1.00–4.10)
No casual male partners	84 (66)		1.92 (1.07––3.45)*
At the last occasion you had anal sex with a casual male partner, how far in advance did you know you were going to have sex?			
One day or more	25 (49)	–	–
Less than an hour/a few hours	64 (53)	–	–
Did not know in advance	91 (75)		
No anal sex with casual partners	84 (66)		
Personal experience using condoms			
Negative/neutral	211 (61)	1	1
Positive	51 (68)	1.21 (0.64–2.27)	1.20 (0.64–2.23)
Confidence in discussing condom use with partners			
Not confident/neutral	77 (60)	1	1
Confident	190 (63)	0.99 (0.58–1.71)	1.00 (0.571.74)
Last tested for HIV			
In the last 6 months	185 (64)	1	1
6 months to 2 years ago	35 (69)	1.20 (0.55–2.59)	1.38 (0.65–2.94)
>2 years/never tested	48 (54)	0.68 (0.37–1.25)	0.64 (0.35–1.17)
Self-perceived risk of HIV			
Unlikely to become HIV+	130 (59)	1	1
Likely to become HIV+	128 (69)	1.82 (1.10–3.02)*	1.84 (1.12–3.04)*
If I was taking PrEP, I would be concerned that people will think I’m HIV positive			
Disagree	160 (70)	1	1
Agree	110 (54)	0.83 (0.50–1.37)	0.78 (0.47–1.28)
Concern about using PrEP because of side-effects and long-term use			
Not concerned	180 (72)	1	1
Concerned	86 (48)	0.35 (0.21–0.59)**	0.37 (0.22–0.62)**
Missing data excluded, may not total *N* = 434.			

*p<0.05, **p<0.005.

## Discussion

This is the first study to assess the willingness to use PrEP among any key population in Myanmar. We found that 62% of the HIV undiagnosed GMT surveyed were classified as willing to use PrEP. The proportion of GMT willing to use PrEP in our study is higher than that reported in studies in Thailand [23–25] and similar to that reported in China [17–19]. Willingness to use PrEP was associated with indicators of sexual risk, self-perceived risk of HIV acquisition, and concern regarding side effects and long-term use of PrEP. The results of this study provide important insights into the acceptability of PrEP as part of a combination HIV prevention approach for this population in Myanmar, and provide a basis from which to explore ongoing ethical, feasibility and cost-effectiveness challenges for PrEP implementation in Myanmar. While male-to-male sex remains criminalized in Myanmar, the most recent National Strategic Plan on HIV and AIDS recognizes the importance of targeted interventions for men who have sex with men and transgender persons [35]. Although legislated criminalization of male-to-male sex is rarely acted upon by law enforcement, ongoing stigma and discrimination constitutes a potential barrier to future PrEP programmes in Myanmar.

The substantial proportion of GMT classified as willing to use PrEP and the relationship between willingness and markers of HIV risk indicate that PrEP would be an effective future HIV prevention option in Myanmar. While sampling in this study through existing service contacts in the two major cities in Myanmar limits the generalizability of these results, assessing willingness in an urban and service-engaged population is highly relevant to informing future implementation programmes in Myanmar. The Burnet Institute/MBCA outreach programme through which participants were recruited has commenced a fixed-site and outreach HIV testing service and treatment referral programme, with most GMT testing reporting previous engagement with the programme’s peer outreach education team. Best practice PrEP implementation will need to occur through regular visits to such services to ensure client safety and to comply with current guidelines. Providing PrEP through such a service would also comply with international guidelines for PrEP provision to occur alongside comprehensive HIV prevention services [38]. Given the concentration of GMT in Yangon and Mandalay, the high prevalence of HIV among MSM in these cities [32], and the existing HIV prevention and clinical services available, the findings in this article are highly relevant for informing the potential for future PrEP implementation in Myanmar.

A substantial proportion of GMT in our study reported sexual behaviours that place them at risk of HIV, including reporting multiple partners and inconsistent condom use. Reporting more than one regular male partner and reporting five or more casual male partners were associated with willingness to use PrEP. In addition, inconsistent condom use was marginally significant and, while not statistically significant due to relatively small participant numbers, GMT in our sample who reported regular sex partners who were HIV positive or of unknown status were more likely to be classified as willing to use PrEP. These results suggesting indicators of sexual risk are associated with willingness to use PrEP are consistent with previous literature [14,16,20,25,26] and with international PrEP eligibility guidelines [38]. The somewhat counter-intuitive finding that GMT reporting not being sexually active in the past three months were more likely to be willing to use PrEP compared with participants reporting one regular or two to five casual partners may reflect future intentions in relation to seeking sexual partners. This finding warrants further investigation but may underscore the importance of disseminating information about PrEP and other prevention options beyond those explicitly reporting HIV risk practices.

Consistent with previous literature [15,16,19,26], concern regarding side effects and long-term use was negatively associated with willingness to use PrEP. This result suggests that potential future implementation of PrEP in Myanmar must also be accompanied by community engagement and education about PrEP, how PrEP is clinically managed and the relative risk of side effects. Also consistent with previous literature [14,15,20,26,39], those subjectively perceiving themselves as at heightened risk of HIV were more likely to report willingness to use PrEP. Current guidelines regarding PrEP eligibility are risk-based [40], and community engagement and education programmes for PrEP implementation in Myanmar offer an opportunity to enhance knowledge regarding risk and risk perception more generally and in the context of assessing PrEP eligibility.

There are number of limitations to our findings. As mentioned earlier, our convenience and snowball sample of GMT engaged in Burnet Institute/MCBA HIV prevention programmes may be not be representative of the broader GMT population in Myanmar and may have biased estimates of willingness to use PrEP. Nevertheless, this service-engaged population is likely to be a target population of future PrEP programmes in Myanmar, and thus, determining the level of willingness to use PrEP among this sample provides a useful initial snapshot of interest in PrEP. The use of HIV prevention peer educators to recruit our sample and administer the survey may have also resulted in a degree of social desirability bias, particularly sexual risk domains. However, peer educators were chosen to recruit participants and administer the survey to improve participation rates. Very low levels of awareness of PrEP may have biased estimates of willingness to use PrEP, relative to other studies in settings where PrEP awareness is generally higher. Increased community awareness of and trust in the evidence supporting the safety and effectiveness of PrEP may increase PrEP acceptability among GMT in Myanmar.

While reported condom use was relatively high in our sample with over two thirds reporting using condoms over 50% of the time, routine surveillance data suggests declining use of condoms among MSM in Myanmar [35,41]. PrEP programmes targeting MSM who report inconsistent condom use therefore offer a potentially important future combination HIV prevention strategy in Myanmar. Ultimately, the cost effectiveness of PrEP within such a strategy will depend heavily on future PrEP pricing. Generic manufacturers of PrEP medications have been retailing PrEP in some Asian markets at approximately 1 USD per month [42,43]. While this is likely to still represent an unfeasible price for PrEP scale-up in Myanmar, bulk purchasing of generic versions and the potential for negotiating compassionate pricing arrangements for low- and middle- income countries [44–46] may make PrEP implementation in Myanmar feasible and cost-effective in the future. Cost-effectiveness of national roll-out of PrEP programmes in Myanmar should be assessed using local real-world costing data and include the costs of appropriate clinical monitoring of potential PrEP side effects. Myanmar’s National Strategic Plan on HIV and AIDS (2016–2020) identifies a feasibility and cost-effectiveness study of PrEP that includes the modelling of PrEP effectiveness to reduce new HIV infections as a priority intervention area [35]. In the medium term, efforts towards health system strengthening and clinical management capacity building will allow for the future implementation of PrEP trials, among other emerging biomedical interventions (including expanded access to HIV treatment and hepatitis C treatment).

## Conclusions

While our findings suggest high PrEP acceptability among GMT in Myanmar, there are a number of implementation challenges that need be considered in resource-limited settings. The provision of PrEP needs to be considered within the context of low coverage of antiretroviral treatment for HIV in some countries [32]. Efforts to secure compassionate pricing for antiretrovirals [44–46] and ethical and effective allocative resourcing [47] to improve both treatment and prevention coverage will be crucial to support the future PrEP expansion in low-resource settings. The feasibility of providing PrEP through national and international non-government organizations must be assessed as this is the most likely setting for PrEP provision to key populations. Through this, the availability of suitably trained clinical workforces, appropriate clinical oversight at these sites and local laboratory capacity to monitor PrEP safety must be explored. The use of trained peers as lay healthcare staff should also be explored as a task-shifting option to relieve healthcare professionals from certain tasks provide an acceptable and “safe” service delivery model for GMT in Myanmar. In particular, future PrEP implementation sites in Myanmar must also be well-equipped to provide acceptable counselling to promote adherence and additional primary prevention options to GMT. Demonstration projects exploring these implementation considerations would be the next step for PrEP in Myanmar.
