# Corrigendum

**DOI:** 10.7448/IAS.20.1.22284

**Published:** 2017-01-01

Regarding the paper, ‘Willingness to use HIV pre‐exposure prophylaxis among gay men, other men who have sex with men and transgender women in Myanmar’ by Bridget L. Draper, Freya J. I. Fowkes, Zaw Min Oo, Zaw Win Thein, Poe Poe Aung, Vanessa Veronese, Claire Ryan, Myo Thant, Chad Hughes, Mark Stoové.

Published in *Journal of the International AIDS Society*, Citation: Draper BL et al. *Journal of the International AIDS Society* 2017, **20**:21885. http://www.jiasociety.org/index.php/jias/article/view/21885 |http://dx.doi.org/10.7448/IAS.20.1.21885

The co‐author Freya J. I. Fowkes was missing from the original author list. The correct author list and information is as follows:

Bridget L. Draper^1§^, Freya J. I. Fowkes^5^, Zaw Min Oo^2^, Zaw Win Thein^2^, Poe Poe Aung^2^, Vanessa Veronese^1,3^, Claire Ryan^2^, Myo Thant^4^, Chad Hughes^1,3^, Mark Stoové^1^

Affiliations:

1 Department of Disease Elimination Burnet Institute, Melbourne, Australia

2 Department of International Development, Burnet Institute, Yangon, Myanmar

3 Department of Epidemiology and Preventive Medicine, Monash University, Melbourne, Australia

4 Yangon Regional Public Health Department, Yangon, Myanmar

5 Centre for Epidemiology and Biostatistics, Melbourne School of Population and Global Health, The University of Melbourne, Melbourne, VIC, Australia.

No competing interests. FJIF contributed to the data cleaning and analysis process.

Updated Authors’ contribution:

ZWO, ZWT, PPA, CR, MT, CH and MS all contributed to the development of the data collection tools, training of peer educators to recruit participants, oversight of recruitment, data collection and interpretation of results. BLD, FJIF, VV and MS led the data cleaning and analysis process. BLD led the writing of the manuscript. All authors have read and approved the final manuscript.

